# *CpSmt3*, an ortholog of small ubiquitin-like modifier, is essential for growth, organelle function, virulence, and antiviral defense in *Cryphonectria parasitica*

**DOI:** 10.3389/fmicb.2024.1391855

**Published:** 2024-05-09

**Authors:** Shuangcai Li, Fengyue Chen, Xiangyu Wei, Luying Yuan, Jiayao Qin, Ru Li, Baoshan Chen

**Affiliations:** ^1^State Key Laboratory for Conservation and Utilization of Subtropical Agro-bioresources, Guangxi Research Center for Microbial and Enzyme Engineering Technology, College of Life Science and Technology, Guangxi University, Nanning, China; ^2^Guangxi Key Laboratory of Sugarcane Biology, College of Agriculture, Guangxi University, Nanning, China

**Keywords:** *Cryphonectria parasitica*, CpSmt3, SUMOylation, virulence, hypovirus

## Abstract

**Introduction:**

SUMOylation is an important post-translational modification that regulates the expression, localization, and activity of substrate proteins, thereby participating in various important cellular processes such as the cell cycle, cell metabolism, gene transcription, and antiviral activity. However, the function of SUMOylation in phytopathogenic fungi has not yet been adequately explored.

**Methods:**

A comprehensive analysis composed of proteomics, affinity pull-down, molecular and cellular approaches was performed to explore the roles of SUMOylation in *Cryphonectria parasitica*, the fungal pathogen responsible for chestnut blight.

**Results and discussion:**

*CpSmt3*, the gene encoding the SUMO protein CpSmt3 in *C. parasitica* was identified and characterized. Deletion of the *CpSmt3* gene resulted in defects in mycelial growth and hyphal morphology, suppression of sporulation, attenuation of virulence, weakening of stress tolerance, and elevated accumulation of hypovirus dsRNA. The Δ*CpSmt3* deletion mutant exhibited an increase in mitochondrial ROS, swollen mitochondria, excess autophagy, and thickened cell walls. About 500 putative SUMO substrate proteins were identified by affinity pull-down, among which many were implicated in the cell cycle, ribosome, translation, and virulence. Proteomics and SUMO substrate analyses further revealed that deletion of *CpSmt3* reduced the accumulation of CpRho1, an important protein that is involved in TOR signal transduction. Silencing of *CpRho1* resulted in a phenotype similar to that of Δ*CpSmt3*, while overexpression of CpRho1 could partly rescue some of the prominent defects in Δ*CpSmt3*. Together, these findings demonstrate that SUMOylation by CpSmt3 is vitally important and provide new insights into the SUMOylation-related regulatory mechanisms in *C. parasitica*.

## 1 Introduction

Small ubiquitin-related modifier (SUMO) is an important protein that modifies substrate proteins and thereby plays a crucial role in various biological processes such as the cell cycle, secondary metabolism, transcription, DNA damage repair, and other cellular processes (Eifler and Vertegaal, 2015; Yau et al., 2021). The process of SUMOylation is similar to that of ubiquitination of a protein. First, the precursor of the SUMO molecule is cleaved at the C-terminus by SUMO protease, exposing the di-glycine motif. With the participation of ATP, the mature SUMO molecule is then connected to the cysteine residue of the E1 enzyme (composed of Aos1 and Uba2), forming a high-energy thioester bond and activating the SUMO molecule. The SUMO molecule is then transferred to the cysteine residue of the E2 enzyme (Ubc9) to form a second thioester bond between SUMO and the E2 enzyme. The E2 enzyme then recognizes the SUMO consensus motif of the target protein and the E3 SUMO ligase facilitates the transfer of the activated SUMO to a lysine residue of the target protein (Chang and Yeh, 2020; Vertegaal, 2022). Although most E3 ligases (Siz1, Siz2, Mms21, and Zip3) are characterized by an SP-RING domain (Cheng et al., 2006; Reindle et al., 2006; Pasupala et al., 2012; Kim et al., 2016), some E3 ligases do not have this canonical domain for catalytic activity (Johnson and Gupta, 2001; Kahyo et al., 2001; Pichler et al., 2002; Kagey et al., 2003).

Recent studies indicate that protein SUMOylation is important for fungi. In *Saccharomyces cerevisiae*, SUMOylation is involved in DNA replication, hypoxia, and protein folding. Disruption of the SUMO-encoding gene *Smt3* results in a lethal phenotype (Takahashi et al., 1999; Newman et al., 2017). In model filamentous fungus *Aspergillus nidulans*, SUMO protein is non-essential for vegetative growth, but required for cellular differentiation (Gupta et al., 2020). In the phytopathogenic fungus *Aspergillus flavus*, deletion of the SUMO gene adversely affected colony forming ability and pathogenicity (Nie et al., 2016).

*Cryphonectria parasitica* is the fungal pathogen responsible for the chestnut blight (Alfen, 1982). It can be infected by *Cryphonectria* hypovirus 1 (CHV1)-EP713. The virus–fungus interaction regulates fungal virulence and virulence-associated traits so the virus could serve as a potent biocontrol agent (Nuss, 2005). The virus exploits the trans-Golgi network and ribosomes of *C. parasitica* for replication. Viral infection alters its host’s physiological processes by regulating the MAPK pathway, the citric acid synthesis pathway, and cellular glutamate biogenesis to bring about a cellular environment that is conducive to viral survival and replication (Jacob-Wilk et al., 2006; Yao et al., 2013; So et al., 2017; So and Kim, 2017; Chiba et al., 2018; Chun et al., 2020). However, the impact of SUMOylation on the virus–fungus interaction has not been investigated.

In this study, we characterized the SUMO protein ortholog CpSmt3 in *C. parasitica*. CpSmt3 was found to be involved in mycelial growth, hyphal morphology, sporulation, organelle function, stress response, virulence, and antiviral defense. Among the proteins that interact with CpSmt3 *in vivo*, CpRho1 seemed to be an essential target of CpSmt3, as strains with silenced *CpRho1* had a phenotype very similar to that of the Δ*CpSmt3* deletion mutant. Collectively, these results demonstrate the key role of SUMOylation in *C. parasitica* growth and provide insights into the mechanisms underlying the diverse functions of SUMOylation in *C. parasitica*.

## 2 Materials and methods

### 2.1 Strains and culture conditions

The *C. parasitica* wildtype strain EP155 (ATCC 38755), its isogenic hypovirus (CHV1-EP713)-infected strain EP155/CHV1-EP713 (designated EP155/+virus; ATCC 52571) (Chen et al., 1994), highly efficient homologous recombinant strain KU80 (Δ*Cpku80* of EP155) (Lan et al., 2008), and derived mutant strains were incubated on potato glucose agar (PDA) medium at 26°C with a 12/12-h light/dark cycle for phenotypic analyses. PDA medium was also used for DNA, RNA, and protein extraction, as previously described (Li et al., 2022).

### 2.2 Generation of gene deletion, complementation and overexpression strains

The Δ*CpSmt3* deletion mutant was constructed using a homologous recombination method based on the KU80 strain. Using EP155 genome DNA as the template, the upstream (1,051 bp) and downstream (1,150 bp) regions flanking the *CpSmt3* gene were amplified using the primers smt3-left-F/smt3-left-R and smt3-right-F/smt3-right-R, respectively. The hygromycin B resistance gene *hph* was amplified using the primers hph-F/hph-R, and then the three amplification products were ligated by fusion polymerase chain reaction (PCR) to obtain a 4.1-kb cassette. The purified products were transformed into KU80 protoplasts mediated by polyethylene glycol (PEG), which were regenerated and screened on medium containing 30 μg/mL hygromycin B (Thermo Fisher Scientific, USA). The transformed strains were consecutively cultured on PDA containing hygromycin B for three generations, and were subsequently identified. According to the standard protocol described by Sambrook and Russell (2001), Southern blot and PCR methods were used to identify the Δ*CpSmt3* deletion mutant. The construction of Δ*CpUbc2* and Δ*CpUbc9* deletion mutants were performed using the same protocol with corresponding primer sets.

The Δ*CpSmt3*-Com complementation strain was generated by amplifying the entire *CpSmt3* gene (open reading frame and promoter sequence) by PCR. Subsequently, the amplified fragment was cloned into the transformation vector pCPXG418, with a geneticin resistance (G418) cassette. The resulting construct pCPXG418-*CpSmt3* was transformed into Δ*CpSmt3*. Δ*CpSmt3*-Com was then confirmed by PCR using primer smt3-qPCR-F/smt3-qPCR-R.

To construct the *CpSmt3*-OE overexpression strain, the *CpSmt3* open reading frame was amplified and then inserted into the vector pCPXG418, with the *gpd* promoter for the transgene. The resulting construct pCPXG418-*CpSmt3* was then introduced into wildtype (EP155) protoplasts. Positive clones were confirmed by PCR and quantitative reverse transcription PCR (qRT-PCR). Δ*CpSmt3* overexpressing *CpRho1* (designated Δ*CpSmt3*/*CpRho*1-OE) was constructed using the same protocol with a corresponding primer set. Primers used in this study are compiled in Supplementary Table 1.

### 2.3 Construction of RNA interference (RNAi) strains

*CpRho1* fragment (including 182 bp intron and 412 bp exon) was amplified by PCR and cloned into the short hairpin RNA (shRNA) expression vector WRNAIPG (constructed by our group), between the Pgpd promoter and TrapC terminator, with hygromycin B resistance as the selection marker. The RNAi construct was then transformed into wildtype (EP155) protoplasts, and confirmed by qRT-PCR. Primers used in this study are compiled in Supplementary Table 1.

### 2.4 Characterization of fungal phenotype

Phenotypic traits (growth rate, pigmentation, sporulation, and stress tolerance) were assessed using previously established methods (Kim et al., 1995). Briefly, for assessing sporulation, fungal strains were cultured on PDA medium at 26°C for 14 days. Conidia were collected and quantified using a hemacytometer. For assessing stress tolerance, strains were cultured on PDA media supplemented with stress chemicals. Three independent replicates were performed for each experiment. For mycelium dry weight assays, the same size blocks (0.5 mm^2^× 0.5 mm^2^) cut from 5-day-old PDA cultures were inoculated into EP liquid medium (Puhalla, 1971). After incubation at 26°C for 7 days, the mycelia in EP liquid medium were filtered and washed. Then, the mycelium was dried at 60°C for 48 h. The dry weight of each mycelium was determined by an electronic balance (Sartorius, Germany).

### 2.5 Microscopy

Light microscopy was performed using an Olympus BX51 fluorescent microscope (Olympus, Japan). Transmission electron microscopy (TEM) was performed using a JEM-1400-FLASH Transmission Electron Microscope (JEOL, Japan). For *in situ* mitochondria analysis, fungal hyphae were cultured in PDA medium for 10 days, scraped, and fixed with 2.5% glutaraldehyde in 100 mM phosphate buffer (pH 7.2) at 4°C overnight. After rinsing with phosphate buffer (50 mM, pH 6.8), the samples were dehydrated, embedded with Epon 812, then sliced into ultrathin sections, stained with uranium acetate (2%), and poststained with lead citrate (Shi et al., 2019). Three biological replicates were performed for each sample.

### 2.6 Assessment of mitochondrial membrane potential (MMP)

Mitochondrial membrane potential was assessed using a JC-1 fluorescent probe (Beyotime, China), and the ratio of JC-1 red/green fluorescence intensity was utilized as a representation of MMP. Briefly, the strains were cultured on PDA medium with a piece of cellophane for 10 days. The harvested mycelia were placed in JC-1 staining buffer containing 10 μM JC-1, incubated at 37°C in the dark for 20 min, and then washed with JC-1 washing buffer twice. The red fluorescence of JC-1 dimers represents normal MMP, while the green fluorescence of JC-1 monomers signifies a depolarized MMP. A transition from red to green fluorescence indicates a reduction in MMP.

### 2.7 Virulence assays

The virulence of the strains was analyzed using dormant stems of Chinese chestnut (*Castanea mollissima*), as described previously (Shi et al., 2014). Canker sizes were measured and photographed 28 days post inoculation. The virulence assay was repeated three times for each fungal strain.

### 2.8 Detection of reactive oxygen species (ROS)

Total fungal ROS production was detected by 2′, 7′-dichlorodihydrofluorescein diacetate (DCFH-DA) staining. The fungal mycelia were incubated with 10 μM DCFH-DA (Beyotime, China) in the dark at 37°C for 30 min, and then washed with PBS (10 mM, pH 7.5) three times. At a maximum excitation wavelength of 480 nm and a maximum emission wavelength of 525 nm, fluorescence microscopy was used to detect fluorescence signals. For mitochondrial ROS (mtROS) level detection, MitoSOX red mitochondrial superoxide dismutase indicator (MEC, USA) was used as described previously (Luo et al., 2021). Briefly, mycelia collected from PDA medium were washed three times with PBS, incubated with 10 μM MitoSOX red for 30 min at room temperature, washed three times with PBS again, and observed using fluorescence microscopy.

### 2.9 Purification of SUMO conjugates

To obtain SUMO conjugates, a plasmid expressing 3 × FLAG–CpSmt3 fusion protein was constructed and transformed into the wildtype strain, and transformants were identified based on G418 resistance. A transformant was confirmed by western blot analysis using an anti-FLAG antibody (1: 2000, ABclonal) as the primary antibody. The verified strain was incubated in liquid EP medium for 3 days then harvested. Total protein was extracted from the cells using NP-40 Lysis Buffer (Beyotime, China) with 1 mM phenylmethylsulfonyl fluoride (PMSF), incubated with anti-FLAG antibody (ABclonal) overnight at 4°C and then mixed with protein A/G agarose (Sangon, China) for 2 h, which was then washed using 1× immunoprecipitation (IP) buffer and 1 × 0.1 IP buffer three and six times, respectively. Next, the lysates were incubated with loading buffer at 95°C for 5 min and then centrifuged at 25°C and 1,200 rpm for 1 min. The proteins were collected, digested using trypsin, and analyzed by LC-MS/MS (Q Exactive HF, Thermo Scientific). The resulting MS/MS data were used to search against the nonredundant *C. parasitica* protein database of the Joint Genome Institute (JGI).^1^ Gene Ontology (GO) and Kyoto Encyclopedia of Genes and Genomes (KEGG) enrichment analyses were performed using eggnog-mapper^2^ and Tbtools software (Chen et al., 2020).

### 2.10 Proteomics analysis

Fungal strains were cultured on PDA medium with a piece of cellophane for 10 days, and then the mycelium was collected for proteome sequencing. Protein isolation was conducted using a fungal protein extraction kit (ProExcell™, China). Next, 20 μg protein was digested overnight using 1 μg sequencing-grade trypsin (Promega Corp., USA). Thereafter, the peptides were prepared for nano-LC-MS/MS by C18 Zip-Tip purification according to the manufacturer’s protocol (Millipore Inc., USA). Three biological replicate samples were then suspended in water with 0.1% formic acid (v/v) and subjected to nano-LC-MS/MS (Q Exactive HF, Thermo Scientific, USA). Briefly, 1 μg peptide sample was injected into a reverse-phase BEH C18 column (100 μm × 100 mm; particle size: 1.7 μm; pore size: 300 Å) (Waters Corp., Massachusetts, USA) for LC using a Waters nanoACQUITY LC system. Peptides eluting from the column were analyzed by data-dependent MS/MS on a Q-Exactive Orbitrap mass spectrometer (Thermo Fisher Scientific Inc., Massachusetts, USA). The data were searched against the *C. parasitica* genome database and a decoy database using the Sequest HT search engine in Proteome Discoverer 1.4 software (Thermo Fisher Scientific Inc., Massachusetts, USA).

Differentially expressed proteins (DEPs) between the wildtype strain and Δ*CpSmt3* were determined using the *t*-test function in R language, with fold change > 1.2 and *p* < 0.05 indicating statistical significance. The number of DEPs was then calculated. GO enrichment analysis of the DEPs was conducted using DAVID software,^3^ with *p* < 0.05 indicating statistical significance. DEPs were also annotated using KEGG Mapper.^4^ The KEGG enrichment analysis of the DEPs was conducted using DAVID software.^5^ Fisher’s exact test was employed as the statistical test, with *p* < 0.05 indicating statistical significance.

## 3 Results

### 3.1 Identification of *CpSmt3* gene in *C. parasitica*

The SUMO ortholog of *C. parasitica* was identified by searching the *C. parasitica* genome database^6^ using the *S. cerevisiae* Smt3 protein sequence as a query (GenBank: QHB07964). A protein comprising 106 amino acids, designated CpSmt3 (JGI ID: 356715), was found to share 68.3% similarity with the *S. cerevisiae* Smt3. The *CpSmt3* gene contains three exons and two introns (Supplementary Figure 1A). Domain analysis revealed that it has a conserved Ubl_Smt3_like domain (coordinates 25–97). Phylogenetic analysis showed that CpSmt3 is most closely related to the SUMO proteins of *Valsa mali* and *Neurospora crassa* (Supplementary Figure 1B).

### 3.2 CpSmt3 is required for fungal development, stress response, and virulence

**FIGURE 1:**
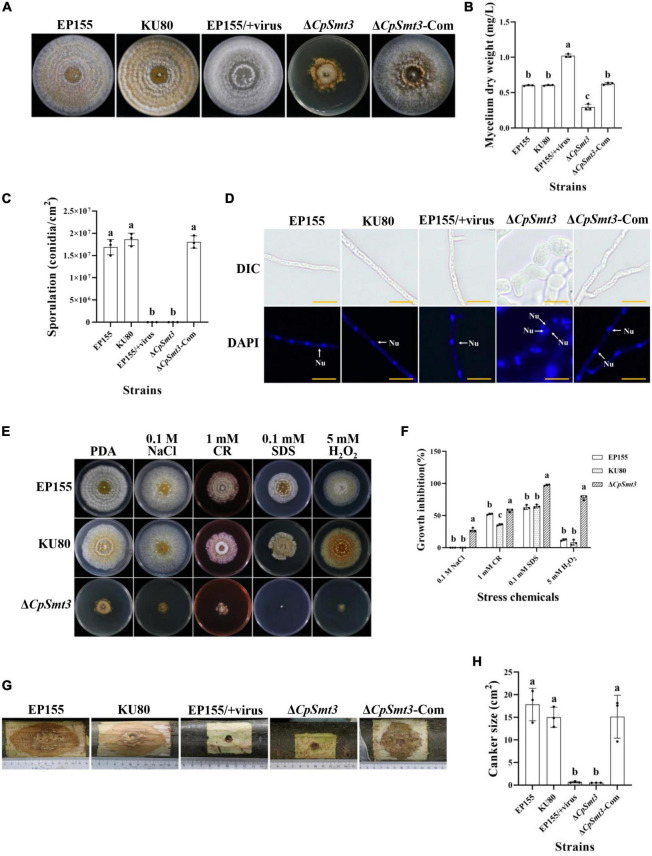
Analysis of colony phenotypes, sporulation, stress tolerance and virulence of *CpSmt3* deletion mutants. **(A)** Colonial morphologies of EP155, KU80, EP155/CHV1-EP713, Δ*CpSmt3*, and Δ*CpSmt3*-Com strains cultured on PDA for 14 days. **(B)** Mycelium dry weight statistics of the tested strains in **(A)**. **(C)** Sporulation levels of the tested strains. **(D)** DAPI staining indicates aberrant nuclear segregation in Δ*CpSmt3* mutant. White arrow represents nucleus. Scale bar = 10 μm. **(E)** Colony morphologies of the tested strains on cultured PDA medium supplemented with stress agents at 26°C for 7 days. **(F)** Colony area statistics of the tested strains in **(E)**. **(G,H)** Cankers induced by the tested strains on dormant stems of Chinese chestnut and statistics of the canker sizes. The inoculated stems were kept at 26°C and cankers were measured and photographed 28 days post inoculation. Error bars represent the standard deviation based on three independent experiments. Different letters on the bars indicate significant differences (*p* < 0.05).

To investigate the function of the *CpSmt3* gene in *C. parasitica*, we constructed a *CpSmt3* deletion mutant (Δ*CpSmt3*) and a *CpSmt3* overexpression strain (*CpSmt3*-OE) (Supplementary Figure 2). Southern blot and RT-PCR analyses of Δ*CpSmt3* showed that *CpSmt3* was successfully deleted from the genome of *C. parasitica* (Supplementary Figures 2B–D). In addition, western blot analysis using anti-SUMO1 antibody showed that the wildtype and complementation strain (Δ*CpSmt3*-Com) had a ∼16 kDa band, while Δ*CpSmt3* did not (Supplementary Figure 2E). This indicated that the CpSmt3 was involved in SUMOylation of *C. parasitica*. Compared to the wildtype strain EP155 and the parental strain KU80, Δ*CpSmt3* exhibited reduced aerial hyphae, intense pigmentation, slow colony growth, and no spore formation (Figures 1A–C). Microscopy showed that the hyphae of Δ*CpSmt3* were swollen and accompanied by multinucleated cells, and the hyphal diameter was also significantly larger than that of the wildtype strain (Figure 1D and Supplementary Figure 2F). However, no significant changes were observed in *CpSmt3*-OE (Supplementary Figures 2G, H).

To determine the role of SUMO in the stress response, wildtype and Δ*CpSmt3* were inoculated on PDA media supplemented with cell stress agents Congo red, sodium dodecyl sulfate (SDS), NaCl, and H_2_O_2_. Δ*CpSmt3* was more sensitive to the stress than the wildtype strain, especially to H_2_O_2_ and SDS, with inhibition rates of 78.02 and 97.69%, respectively (Figures 1E, F).

To explore the role of *CpSmt3* in *C. parasitica* virulence, chestnut stems were inoculated with the wildtype strain, KU80, EP155/+virus, Δ*CpSmt3*, and complementation strain to assess their virulence. The canker formed by Δ*CpSmt3* was significantly smaller than that of wildtype strain or KU80, but comparable to the canker formed by the hypovirus-infected wildtype strain. The reduced virulence of Δ*CpSmt3* was fully restored by re-induction of the wildtype copy of *CpSmt3* (Figures 1G, H).

### 3.3 Deletion of CpSmt3 results in organelle defects and excessed autophagy

**FIGURE 2:**
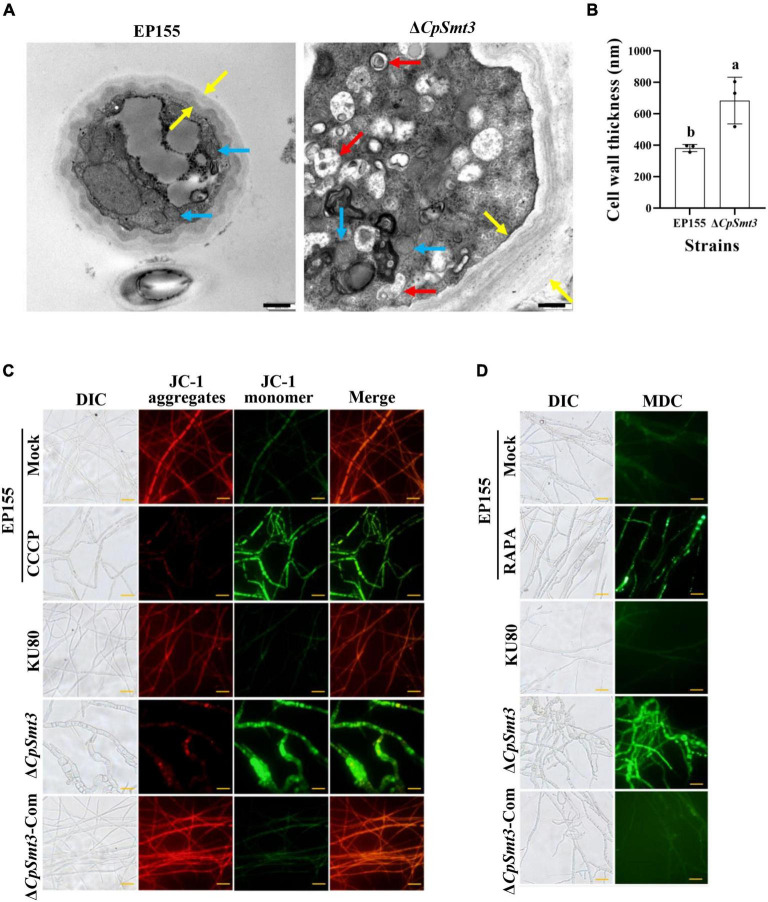
Deletion of *CpSmt3* caused organelle defects and fungal autophagy. **(A)** Morphology of mitochondria, autophagic vacuoles, and cell walls under transmission electron microscope. Blue arrow represents mitochondrion, red arrow represents autophagic vacuole, and yellow arrow represents cell wall. Scale bar = 500 nm. **(B)** Cell wall thickness was measured by ImageJ software. Error bars represent the standard deviation based on three independent experiments. Different letters on the bars indicate significant differences (*p* < 0.05). **(C)** Assay of mitochondrial membrane potential (MMP). Mycelium was subjected to JC-1 staining and viewed using an Olympus fluorescent microscope. Wildtype mycelium treated with 10 μM CCCP for 24 h was used as the positive control. In healthy mitochondria, JC-1 forms a polymer and emits red fluorescence. When MMP decreases, JC-1 is stored as a monomer and emits green fluorescence. **(D)** Assay of autophagy. Mycelium was incubated with MDC at 37°C for 10 min. Wildtype strain treated with 10 nM rapamycin (RAPA) for 48 h was used as the positive control. Autophagic vesicles emit green fluorescence by excitation of ultraviolet light. Scale bar = 20 μm.

As SUMOylation has been reported to be involved in mitochondrial and cell wall morphology (Yamada et al., 2021; Azizullah et al., 2023), we wondered whether deletion of *CpSmt3* would have similar effects in *C. parasitica*. TEM revealed that Δ*CpSmt3* exhibited swollen mitochondria and a thickened cell wall compared to the wildtype strain (Figures 2A, B).

To further explore the mitochondrial dysfunction caused by *CpSmt3* deletion, JC-1 staining was used to measure the MMP. There was a significant decrease in MMP upon *CpSmt3* deletion (Figure 2C). In addition, TEM analysis showed that autophagic bodies were evident in the mycelia of Δ*CpSmt3* (Figure 2A). Monodansylcadaverine (MDC) staining further proved that autophagic bodies accumulated in Δ*CpSmt3* cells, but not in the wildtype strain (Figure 2D). Together, these results suggested that deletion of *CpSmt3* caused organelle defects and autophagy.

### 3.4 Deletion of CpSmt3 causes mitochondrial ROS burst

**FIGURE 3:**
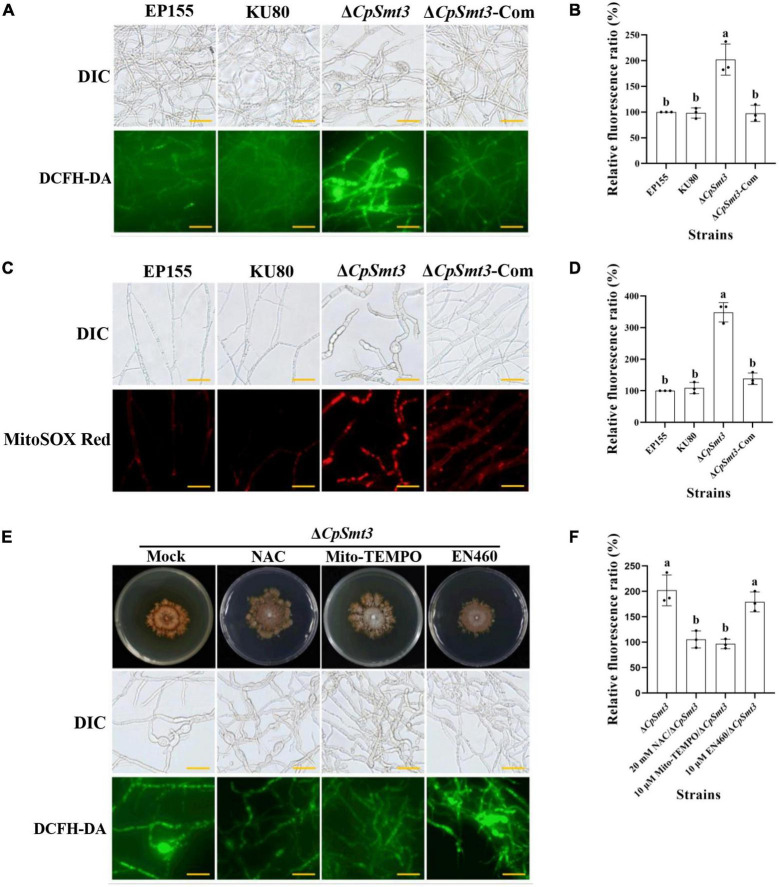
**(A)** ROS production of the tested strains was detected by DCFH-DA staining. Scale bar = 20 μm. **(B)** Relative ROS fluorescence ratio of the tested strains was analyzed by ImageJ software. **(C)** mtROS production of the tested strains was detected by MitoSOX red staining. Red fluorescence indicates mtROS production. Scale bar = 20 μm. **(D)** Relative mtROS fluorescence ratio of the tested strains was analyzed by ImageJ software. **(E)** Changes in ROS production in Δ*CpSmt3* treated with 20 mM NAC, 10 μM Mito-TEMPO, or 10 μM EN460. Scale bar = 20 μm. **(F)** Relative ROS fluorescence of tested strains was analysis by Image J software. Error bars represent the standard deviation based on three independent experiments. Different letters on the bars indicate significant differences (*p* < 0.05).

Previous studies have shown that SUMOylation play essential roles in the regulation of ROS homeostasis by controlling ROS production and clearance, and reduced SUMOylation may lead to higher ROS production (Stankovic-Valentin and Melchior, 2018). As deletion of *CpSmt3* caused decreased MMP, we measured the intracellular ROS in Δ*CpSmt3* using the DCFH-DA fluorescence assay. Compared to the wildtype strain, Δ*CpSmt3* had a significantly higher ROS level (Figures 3A, B). To further identify the source of ROS in Δ*CpSmt3*, we assessed the mtROS by using MitoSOX Red staining. A large amount of mtROS was observed in Δ*CpSmt3* (Figures 3C, D), suggesting that the ROS burst was derived from mitochondria. To confirm this speculation, we supplemented the PDA medium with the ROS inhibitor N-Acetyl-L-cysteine (NAC) or the mtROS-specific inhibitor Mito-TEMPO. After the treatment, the Δ*CpSmt3* colonies became larger and the ROS level was significantly reduced. In contrast, treatment with the ERO1-α inhibitor EN460 failed to increase the Δ*CpSmt3* colony size or reduce the ROS level (Figures 3E, F).

### 3.5 CpSmt3 inhibits CHV1 replication by CpDcl2 upregulation

**FIGURE 4:**
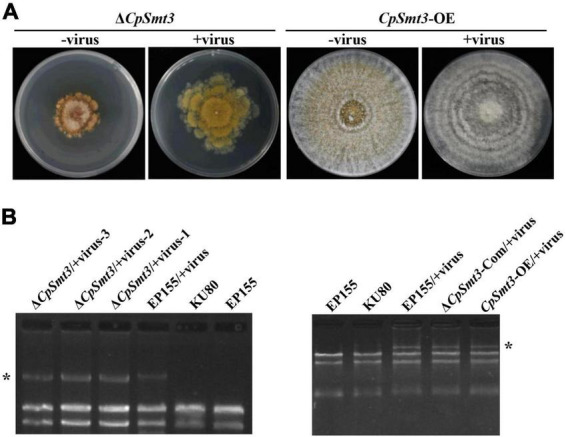
SUMOylation is involved in regulating CHV1 RNA accumulation. **(A)** Colony morphologies of Δ*CpSmt3* and *CpSmt3*-OE, and their CHV1-infected strains. **(B)** Agarose gel electrophoresis of total RNA without gDNA. * indicates position of full-length CHV1 RNA.

In animals, SUMOylation has been reported to activate the immune system to counteract viral infections (Imbert and Langford, 2021). To examine the role of SUMOylation in the interaction between *C. parasitica* and the hypovirus CHV1-EP713, the hypovirus was introduced into Δ*CpSmt3* (Δ*CpSmt3*/CHV1-EP713, designated Δ*CpSmt3*/+virus) via hyphal anastomosis with the isogenic hypovirus-infected wildtype strain (EP155/+virus). The resultant colony size was significantly increased compared to Δ*CpSmt3*, suggesting that hypovirus infection partially reversed the growth impairment resulting from *CpSmt3* deletion. In contrast, the hypovirus-infected *CpSmt3*-OE did not exhibit a change in colony size compared to the hypovirus-infected wildtype strain (Figures 4A and Supplementary Figure 3D). However, when Δ*CpSmt3* and Δ*CpSmt3*/+virus inoculated on chestnut branches, the canker sizes of them were the same (Supplementary Figure 3A). Interestingly, hypovirus-infected Δ*CpSmt3* accumulated about five times more viral dsRNA than the hypovirus-infected wildtype strain, while hypovirus-infected Δ*CpSmt3*-Com regained the ability to inhibit viral dsRNA accumulation. Hypovirus-infected *CpSmt3*-OE did not exhibit an altered viral dsRNA accumulation compared to the hypovirus-infected wildtype strain (Figures 4B and Supplementary Figure 3E).

**FIGURE 5:**
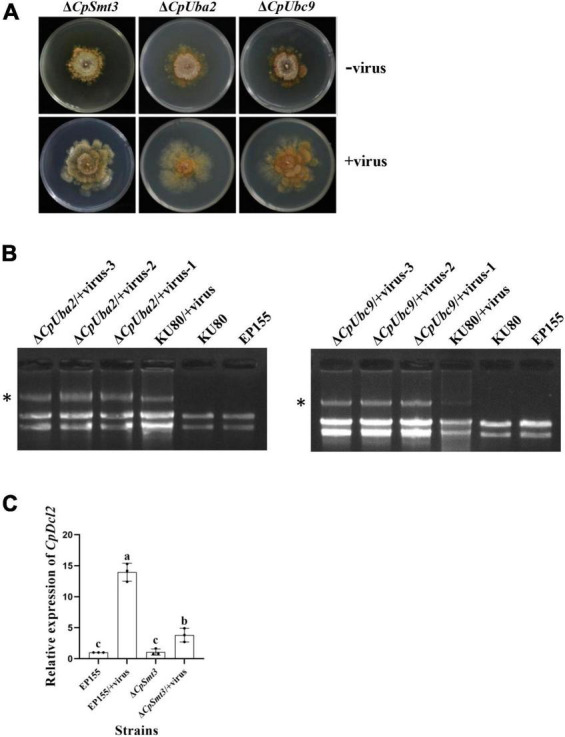
Deletion of *CpUba2* or *CpUbc9* increased CHV1 RNA accumulation. **(A)** Colony morphologies of Δ*CpSmt3*, Δ*CpUba2*, and Δ*CpUbc9* and their CHV1-infected strains. **(B)** Agarose gel electrophoresis of viral dsRNA accumulation in the tested strains. * represents CHV1 RNA. **(C)** qRTPCR analysis of the relative expression of *CpDcl2* gene. The expression of *CpDcl2* in EP155 was set at 1.0, and the levels of indicated strains were expressed as fold change relative to those in EP155. Error bars represent the standard deviation from three independent experiments. Different letters on the bars indicate significant differences (*p* < 0.05).

To verify whether SUMOylation influences hypovirus replication, we constructed SUMO E1 subunit Uba2 and E2 enzyme Ubc9 mutant strains of *C. parasitica*, designated Δ*CpUba2* (JGI ID: 255521) and Δ*CpUbc9* (JGI ID: 324396), respectively (Supplementary Figures 3B, C). They both exhibited a phenotype very similar to that of Δ*CpSmt3*, and hypovirus infection of both strains partially rescued the colony size and growth (Figure 5A and Supplementary Figure 3D) but viral dsRNA accumulation was elevated five- and ten-fold, respectively (Figures 5B and Supplementary Figure 3E).

To investigate the molecular mechanism underlying the increased viral dsRNA, we measured the transcript level of *CpDcl2*, a key gene in the antiviral RNA silencing pathway. There was no significant difference in *CpDcl2* expression between the wildtype strain and Δ*CpSmt3*. However, *CpDcl2* was highly upregulated (13-fold) in the hypovirus-infected wildtype strain, but only upregulated ∼4-fold in the hypovirus-infected Δ*CpSmt3* strain (Figure 5C). These results suggest that SUMOylation is required for strong inhibition of viral dsRNA accumulation, likely by upregulating *CpDcl2* to suppress viral replication.

### 3.6 Deletion of CpSmt3 alters protein expression pattern in *C. parasitica*

It is generally believed that SUMOylation increases protein stability through competitive binding to the ubiquitination modification sites in the substrate proteins (Bae et al., 2004; Prudent et al., 2015; Nakagawa et al., 2016). To explore the molecular mechanism of SUMOylation in phenotype, we performed a label-free proteomic analysis of wildtype vs. Δ*CpSmt3* mycelia. A total of 3,970 and 3,254 proteins were identified in these strains, respectively, representing 35.82% (4,158 proteins) of the 11,609 annotated proteins from the whole genome (Supplementary Table 2). Compared to the wildtype strain, 1,869 differentially expressed proteins (DEPs), 1,702 downregulated and 167 upregulated, were observed in Δ*CpSmt3* (Supplementary Table 2).

**FIGURE 6:**
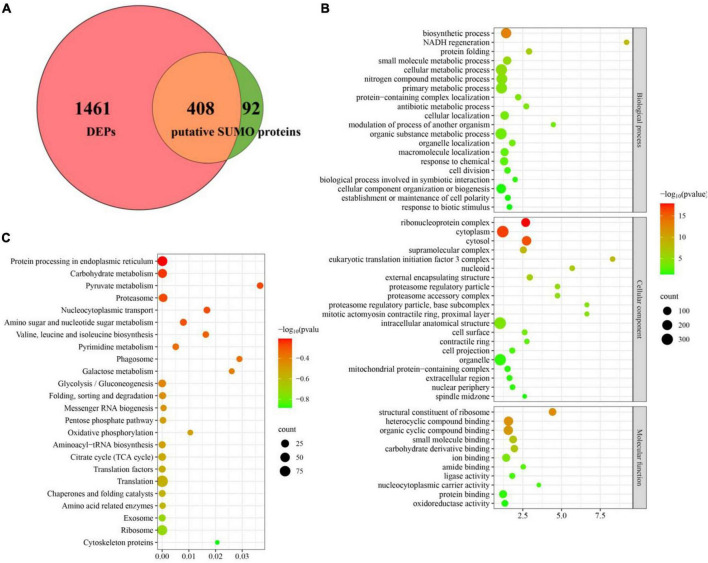
Deletion of *CpSmt3* results in a reduction of total SUMOylated proteins. **(A)** Venn diagram of proteomics and co-immunoprecipitation data. The overlapping orange area indicates that there were 408 SUMOylated proteins whose expression levels changed significantly in the proteomics analysis of Δ*CpSmt3* vs. wildtype strain. **(B)** GO enrichment analysis of 408 overlapping proteins shown in **(A)**. **(C)** KEGG enrichment analysis of 408 overlapping proteins shown in **(A)**. All the pathways shown in maps were significantly enriched (*p* < 0.01).

To define the SUMOylation target spectrum of CpSmt3 in *C. parasitica*, we enriched the SUMOylated proteins in a wildtype (EP155) transformant expressing 3 × FLAG–CpSmt3 fusion protein, using affinity pull-down assays involving anti-FLAG beads. Western blot analysis with anti-FLAG antibody showed that the fusion protein was successfully expressed (Supplementary Figure 4A). The anti-FLAG bead-captured proteins were then trypsin-digested and subjected to LC-MS/MS analysis. A total of 500 SUMOylated proteins were identified in three independent biological replicates. A total of 1,229 SUMOylation sites and 644 SUMO interaction motifs (SIM) were identified within these proteins using the SUMO site prediction software GPS-SUMO 2.0 online tool^7^ (Supplementary Figures 4B, C and Supplementary Table 3). Interestingly, the majority (408 out of 500) of these potential SUMOylated proteins were the DEPs identified in Δ*CpSmt3* (Figure 6A). Intriguingly, most of these SUMOylated DEPs (397 out of 408) were downregulated in Δ*CpSmt3*, including the five pathogenesis-related proteins listed in Supplementary Table 3.

Gene Ontology enrichment analysis indicated that the downregulated SUMOylated proteins in Δ*CpSmt3* were significantly enriched in structural constituent of ribosome, ribonucleoprotein complex, cytoplasm, cytosol, protein folding, and response to stress (Figure 6B and Supplementary Table 4). KEGG enrichment analysis showed that these proteins were significantly enriched in translation, ribosome, exosome, citrate cycle, translation factors, and oxidative phosphorylation pathway (Figure 6C and Supplementary Table 5), implying that SUMOylation is essential for maintaining ribosomal and mitochondrial function and survival in response to environmental stress.

### 3.7 CpRho1 is the key control point for CpSmt3-regulated traits

**FIGURE 7:**
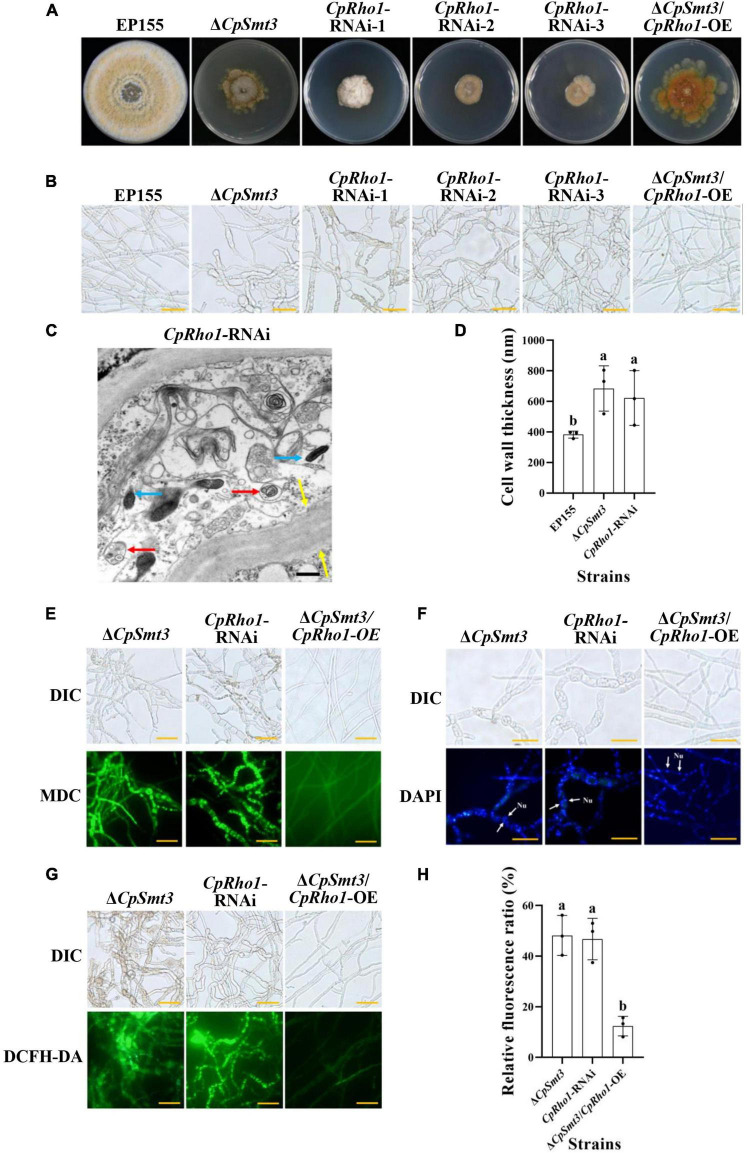
*CpRho1* is essential for growth, organelle function, and the cell cycle. **(A)** Colony morphologies of wildtype strain, Δ*CpSmt3*, *CpRho1* RNA interference strains (*CpRho1*-RNAi), and *CpRho1*-overexpressing Δ*CpSmt3* (Δ*CpSmt3*+*CpRho1*-OE). **(B)** Mycelial morphologies of the tested strains. **(C)** Organelle structure of *CpRho1*-RNAi was observed by TEM. Blue arrow represents mitochondrion, red arrow represents autophagic vacuole, and yellow arrow represents cell wall. Scale bar = 500 nm. **(D)** Cell wall thickness statistics of wildtype, Δ*CpSmt3*, and *CpRho1*-RNAi strains. Cell wall thickness was measured by ImageJ software. **(E)** MDC staining showed autophagy in Δ*CpSmt3* and *CpRho1*-RNAi, but not Δ*CpSmt3*+*CpRho1*-OE. Scale bar = 20 μm. **(F)** DAPI staining showed aberrant nuclear segregation in Δ*CpSmt3* and *CpRho1*-RNAi, but not Δ*CpSmt3*+*CpRho1*-OE. Scale bar = 20 μm. **(G)** DCFH-DA staining showed the ROS burst in Δ*CpSmt3* and *CpRho1*-RNAi, but not Δ*CpSmt3*+*CpRho1*-OE. **(H)** Relative ROS fluorescence ratio of the tested strains in **(G)** measured by ImageJ software. Error bars represent the standard deviation from three independent experiments. Different letters on the bars indicate significant differences (*p* < 0.05).

To further explore the regulated mechanism of CpSmt3, we attempted to find out the proteins related to phenotypic defects of Δ*CpSmt3* mutant from SUMO substrates with known functions (Table 1). We found Rho1 was related to cell cycle, which has also been reported in *Aspergillus fumigatus* that regulates cell wall integrity and stress response to H_2_O_2_ stress (Zhang et al., 2018). In addition, CpRho1 (JGI ID: 97360) was downregulated SUMOylated DEPs in Δ*CpSmt3* (Supplementary Tables 2, 3). Therefore, we selected CpRho1 as the target for further study. To examine the contribution of this protein in *C. parasitica*, we attempted to generate a *CpRho1* deletion mutant. However, we failed to obtain a *CpRho1*-knockout strain after screening hundreds of candidate strains. Thus, we opted to generate a *CpRho1*-silenced strain. Three independent strains, *CpRho1*-RNAi-1, *CpRho1*-RNAi-2, and *CpRho1*-RNAi-3, with 64, 73, and 51% *CpRho1* mRNA levels, respectively (Supplementary Figures 5A, B), were selected for further analysis. They exhibited 84 to 93% reduction in colony size, had no spore formation, and their hyphae were swollen, similar to that of Δ*CpSmt3*. However, *CpRho1* overexpression in Δ*CpSmt3* (Δ*CpSmt3*/*CpRho*1-OE) partially restored the reduced colony size of Δ*CpSmt3* (Figures 7A, B and Supplementary Figure 5C), suggesting a link between CpSmt3 and CpRho1 in modulating fungal traits. TEM showed autophagic vacuoles and significantly thickened hyphal cell walls in the *CpRho1*-RNAi mutants, but the mitochondrial morphology remained normal (Figures 7C, D). Cellular analysis showed that the *CpRho1*-RNAi mutants had increased accumulation of autophagic vacuoles, elevated ROS production, and multinucleation. Importantly, these aberrations were greatly ameliorated by *CpRho1* overexpression in Δ*CpSmt3* (Δ*CpSmt3*/*CpRho1*-OE) (Figures 7E–H), implying that CpSmt3 may exert its effects by maintaining a proper CpRho1 level to ensure the normal cellular functions of the fungus.

**TABLE 1:** The down-regulated SUMOylated proteins with known function in *C. parasitica*.

Protein ID	Functions involved
51523 (SRP14), 34501 (SRP54), 289296 (SRP68), 269583 (SRP72), 84573 (BIP)	Protein export
355736 (eIF2α), 90324 (PPP2C), 99210 (MAPK7), 256639 (SEC18), 277437 (ACTR2)	Autophagy
245397 (CDC46), 354415 (SKP1), 344007 (PPP2R1), 278487 (PPP2R2), 51448 (MAPK1_3), 90324 (PPP2C), 97360 (CpRho1)	Cell cycle
107611 (CpSep1), 99210 (CpSlt2), 79817 (CpMk2), 77211 (CpCdc48), 79817 (CpMk1)	Virulence
292762 (CpAgl2)	Antiviral defense

## 4 Discussion

In fungi, SUMO is encoded by *Smt3* and deletion of this gene leads to alterations in many important phenotypic traits, such as abnormal distribution of chitin in the cell wall, septum formation defect, cell cycle disturbance, impairment of appressorium development, and loss of pathogenicity (Leach et al., 2011; Nie et al., 2016; Wotton et al., 2017; Liu et al., 2018). In this study, we characterized the SUMO-encoding gene, *CpSmt3*, in the chestnut blight fungus *C. parasitica*, and investigated its functions using a gene knockout strategy. CpSmt3 shares 85.1% similarity with Smt3 of the model filamentous fungus *N. crassa* and 68.3% similarity with Smt3 of the yeast *S. cerevisiae*, and it has the characteristic domains of a SUMO protein (Supplementary Figure 1B). Deletion of *CpSmt3* in *C. parasitica* resulted in colony, hyphae, and growth defects, abnormalities of important cellular organelles, and attenuation of virulence and ROS burst (Figures 1–3). In the rice blast fungus *Magnaporthe oryzae*, SUMOylation has been shown to be involved in translation, ribosome biogenesis, the cell cycle, and nuclear division (Liu et al., 2018). Similarly, our comparative proteomics analysis showed that essential cellular processes were significantly altered in Δ*CpSmt3* compared to the wildtype strain (Figure 6 and Supplementary Tables 2–5).

SUMOylation involves a large number of substrate proteins and promotes protein stability by competing with ubiquitin for binding sites, thereby protecting the proteins from degradation (Chang and Yeh, 2020). In line with this, we found that 97% (397 out of 408) of SUMOylated DEPs were downregulated in Δ*CpSmt3* (Figure 6A and Supplementary Table 3). These downregulated proteins were implicated in a wide range of biological processes, including translation, ribosome biogenesis, the cell cycle, oxidative phosphorylation and TCA cycle, protein processing in endoplasmic reticulum, and exosome (Supplementary Tables 4, 5). Abnormalities of these cellular processes jeopardize cell growth and functions, from energy metabolism to pathogenicity (Fernández-Álvarez et al., 2013; Koh and Sarin, 2018; Richardson, 2019; Zhao et al., 2021; Yuan et al., 2023). Of the SUMOylated proteins, CpSep1, CpSlt2, CpMK2, CpMK1, and Cdc48 have been shown to be virulence factors in *C. parasitica* (Park et al., 2004; Choi et al., 2005; Ko et al., 2016; So et al., 2017; Jo et al., 2019). Therefore, we speculate that more SUMOylated proteins may also contribute to the phenotypic traits and virulence of *C. parasitica*.

Rho1, a member of the Rho GTPase family, is a component of the TOR signaling pathway, capable of regulating the CREB phosphorylation and involved in actin polarization and cell wall biosynthesis (Qadota et al., 1996; Drgonová et al., 1999). In mammals, Rho1 regulates mitochondria distribution and function, and the defect of Rho1 can lead to abnormality of oxidative phosphorylation, reduced MMP, and increased ROS production (Minin et al., 2006; Zhang and Jiang, 2017). In the fungus *Schizosaccharomyces pombe*, Rho1 is essential for viability, cell cycle, and cell wall integrity (Drgonová et al., 1999; Zhang et al., 2018; Vicente-Soler et al., 2021). In this study, we showed that CpRho1 was a SUMOylation target and it was significantly downregulated in Δ*CpSmt3*, indicating that CpSmt3 may regulate the stability of CpRho1. As the hyphal morphology, and cell wall thickness of the *CpRho1*-RNAi mutants were similar to those of Δ*CpSmt3*, and *CpRho1* overexpression rescued some prominent defects of Δ*CpSmt3* (Figure 7), we propose that the impact of CpSmt3 on the *C. parasitica* phenotype may largely depend on its regulation of CpRho1, likely by stabilizing the CpRho1 protein. Interestingly, the phenotype of Δ*CpSmt3*/+virus mutant was similar to that of the Δ*CpSmt3*/*CpRho1*-OE mutant, and neither of them had ROS burst or autophagy, indicating that CHV1 infection might rescue the phenotype of the Δ*CpSmt3* mutant by upregulated the expression of *CpRho1*. Thus, CpRho1 is a key control point for regulation of important traits in *C. parasitica*, and this may offer a new opportunity to combat the pathogen by targeting this protein or its pathway.

SUMOylation plays an important role in antiviral immunity. In both mammals and plants, SUMOylation enhances the host immunity against virus infection by regulating immune signaling pathways (Saleh et al., 2015; El Motiam et al., 2020). In *C. parasitica*, *CpDcl2* and *CpAgl2* have been shown to play crucial roles in inhibiting the replication of hypovirus CHV1-EP713 (Segers et al., 2007). In this study, we found that CpAgl2 was a SUMOylation target and it was significantly downregulated in Δ*CpSmt3*. It was sharply upregulated upon hypovirus infection in the wildtype strain but highly downregulated in hypovirus-infected Δ*CpSmt3* (Figure 5C). This discrepancy may compromise the antiviral activity in Δ*CpSmt3* and result in more viral dsRNA accumulation (Figure 4B). These findings suggest that SUMOylation regulates antiviral activity at the transcription level in *C. parasitica*. Relatedly, previous studies have shown that Spt–Ada–Gcn5 acetyltransferase (SAGA), a universal transcriptional coactivator, upregulates *CpDcl2* upon hypovirus infection, and knockout of SAGA subunits Gcn5, Sgf73, and Ada2 abolished any induction of *CpDcl2* transcription upon viral infection (Andika et al., 2017, 2019). As Gcn5 is a SUMOylated protein (Sterner et al., 2006; Espinola-Lopez and Tan, 2021), we hypothesize that SUMOylation of the SAGA complex might be used to modulate resistance to viral infection in *C. parasitica*.

## Data availability statement

The original contributions presented in this study are included in this article/Supplementary material, further inquiries can be directed to the corresponding authors. The data presented in the study are deposited in iPROX (https://www.iprox.cn/), accession number: PXD051810 and (https://www.ebi.ac.uk/pride), accession number: PXD051781.

## Author contributions

SL: Data curation, Formal analysis, Methodology, Software, Writing – original draft. FC: Data curation, Methodology, Software, Writing – review & editing. XW: Data curation, Methodology, Software, Writing – review & editing. LY: Data curation, Methodology, Software, Writing – review & editing. JQ: Data curation, Methodology, Software, Writing – review & editing. RL: Conceptualization, Data curation, Formal analysis, Project administration, Supervision, Writing – review & editing. BC: Conceptualization, Formal analysis, Project administration, Resources, Supervision, Writing – review & editing.

## Footnotes

1
http://genome.jgi-psf.org/Crypa1/Crypa1.home.html

2
http://eggnog-mapper.embl.de/

3
https://david-d.ncifcrf.gov/

4
https://www.kegg.jp/kegg/mapper/

5
https://david.ncifcrf.gov/

6
http://genome.jgi-psf.org/Crypa2/Crypa2.home.html

7
http://sumosp.biocuckoo.org/online.php
