# Supplementary material for: CpSmt3, an ortholog of small ubiquitin-like modifier, is essential for growth, organelle function, virulence, and antiviral defense in Cryphonectria parasitica
Source: Front Microbiol. 2024 May 9;15:1391855. doi: 10.3389/fmicb.2024.1391855 (PMC11111931; doi:10.3389/fmicb.2024.1391855)
Supplement: Supplementary file 1 [file Data_Sheet_1.zip › Supplementary Figures.pdf]

Supplementary Material

1. Supplementary Figures

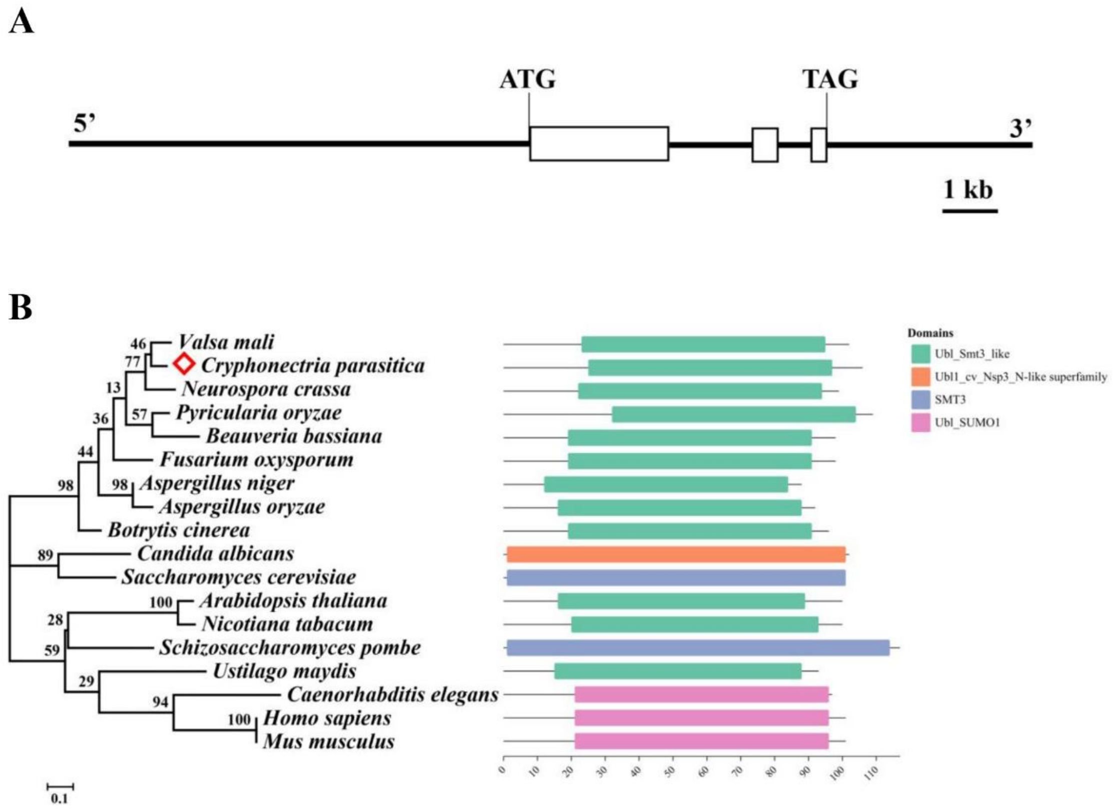

Figure S1. Identification of *CpSmt3* gene. (A) Structure of *CpSmt3* gene. (B) Phylogenetic tree analysis of Smt3 protein. The phylogenetic tree was constructed in MEGA 11.0 software by the maximum-likelihood method. The Smt3 protein domain analyzed by Batch CD-Search tool. (GenBank Accession: *Valsa mal*i (KUI60441), *Neurospora crassa* (ESA43995), *Pyricularia oryzae* (XP_003710648), *Beauveria bassiana* (KAH8708051), *Fusarium oxysporum* (KAJ4278782), *Aspergillus niger* (XP_001392412), *Aspergillus oryzae* (OOO09160), *Botrytis cinereal* (XP_024551758), *Candida albicans* (KAF6062557), *Saccharomyces cerevisiae* (QHB07964), *Homo sapiens* (NP_001005781), *Mus musculus* (NP_033486), *Caenorhabditis elegans* (pdb|5XQM|A), *Schizosaccharomyces pombe* (CAB44758), *Ustilago maydis* (XP_011392223), *Arabidopsis thaliana* (AAN03845), *Nicotiana tabacum* (XP_016456854)).

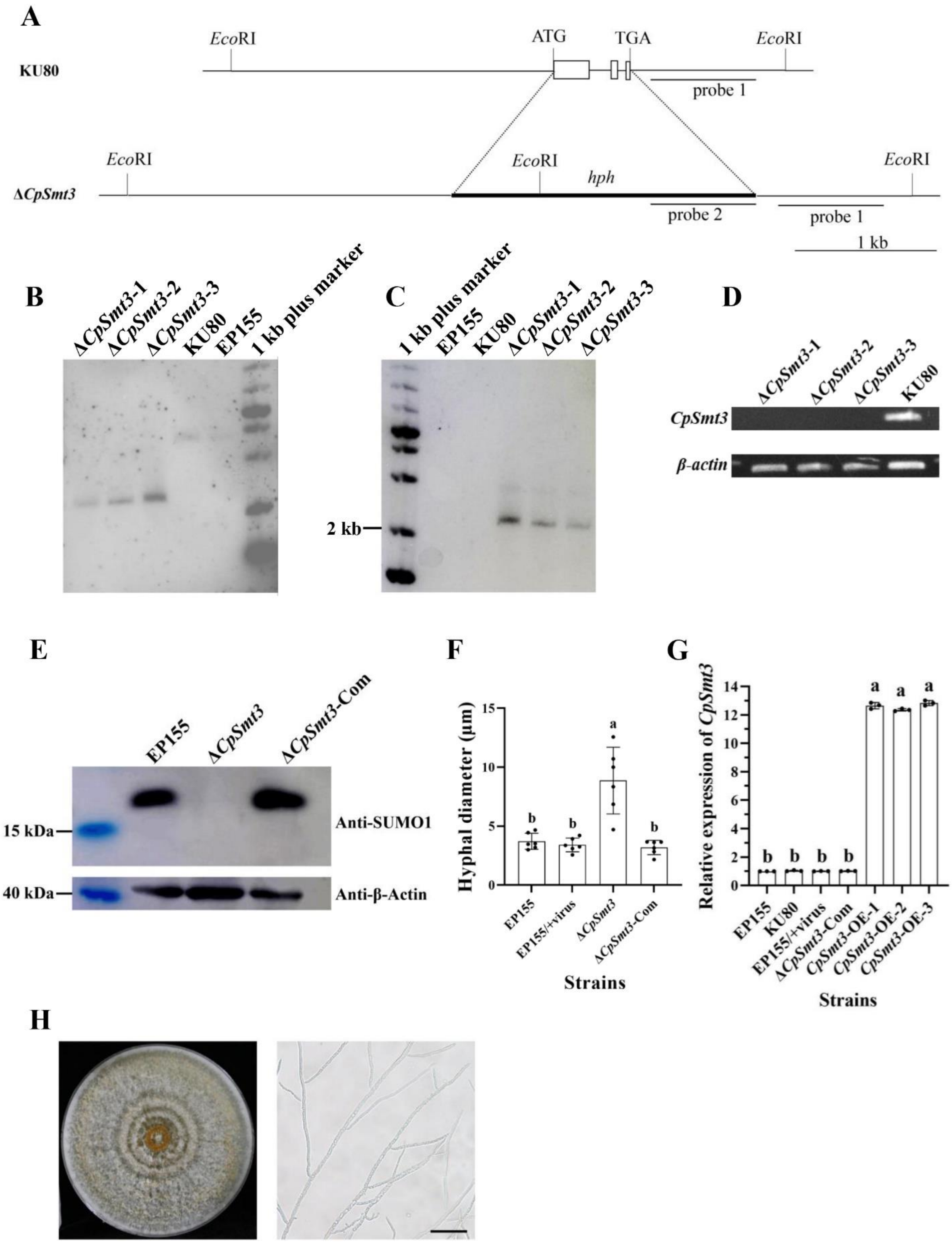
A
EcoRI
ATG
TGA
EcoRI
KU80
probe 1
EcoRI
EcoRI
hph
EcoRI
ΔCpSmt3
probe 2
probe 1
1 kb
B
ΔCpSmt3-1
ΔCpSmt3-2
ΔCpSmt3-3
KU80
EP155
1 kb plus marker
C
1 kb plus marker
EP155
KU80
ΔCpSmt3-1
ΔCpSmt3-2
ΔCpSmt3-3
2 kb
D
ΔCpSmt3-1
ΔCpSmt3-2
ΔCpSmt3-3
KU80
CpSmt3
β-actin
E
EP155
ΔCpSmt3
ΔCpSmt3-Com
15 kDa
Anti-SUMO1
40 kDa
Anti-β-Actin
F
Hyphal diameter (μm)
Strains
EP155
EP155/+virus
ΔCpSmt3
ΔCpSmt3-Com
G
Relative expression of CpSmt3
Strains
EP155
KU80
EP155/+virus
ΔCpSmt3-Com
CpSmt3-OE-1
CpSmt3-OE-2
CpSmt3-OE-3
H

Figure S2. Construction of *CpSmt3* gene deletion and overexpression strains. **(A)** Strategy of *CpSmt3* gene deletion. **(B, C)** Southern blotting analysis of Δ*CpSmt3* mutants, (B) was detected by probe 1, (C) was detected by probe 2. **(D)** Detect the expression of *CpSmt3* gene by RT-PCR, *β-actin* was as internal reference gene. **(E)** CpSmt3 protein expression of EP155, Δ*CpSmt3* mutant, and complement strain was detected by anti-SUMO antibody. β-actin was as internal reference. **(F)** Hyphae diameter statistics showed that deletion of Δ*CpSmt3* resulted in a significant increase in hyphae diameter. **(G)** qRT-PCR analysis of the relative expression of *CpSmt3* gene, the *CpSmt3* gene expression in EP155 strain was set as 1. (H) Colonial and hyphal morphology of the *CpSmt3*-OE strain. Error bars represent the standard deviation from three independent experiments. Different letters on the bars indicate significant differences ($p < 0.05$).

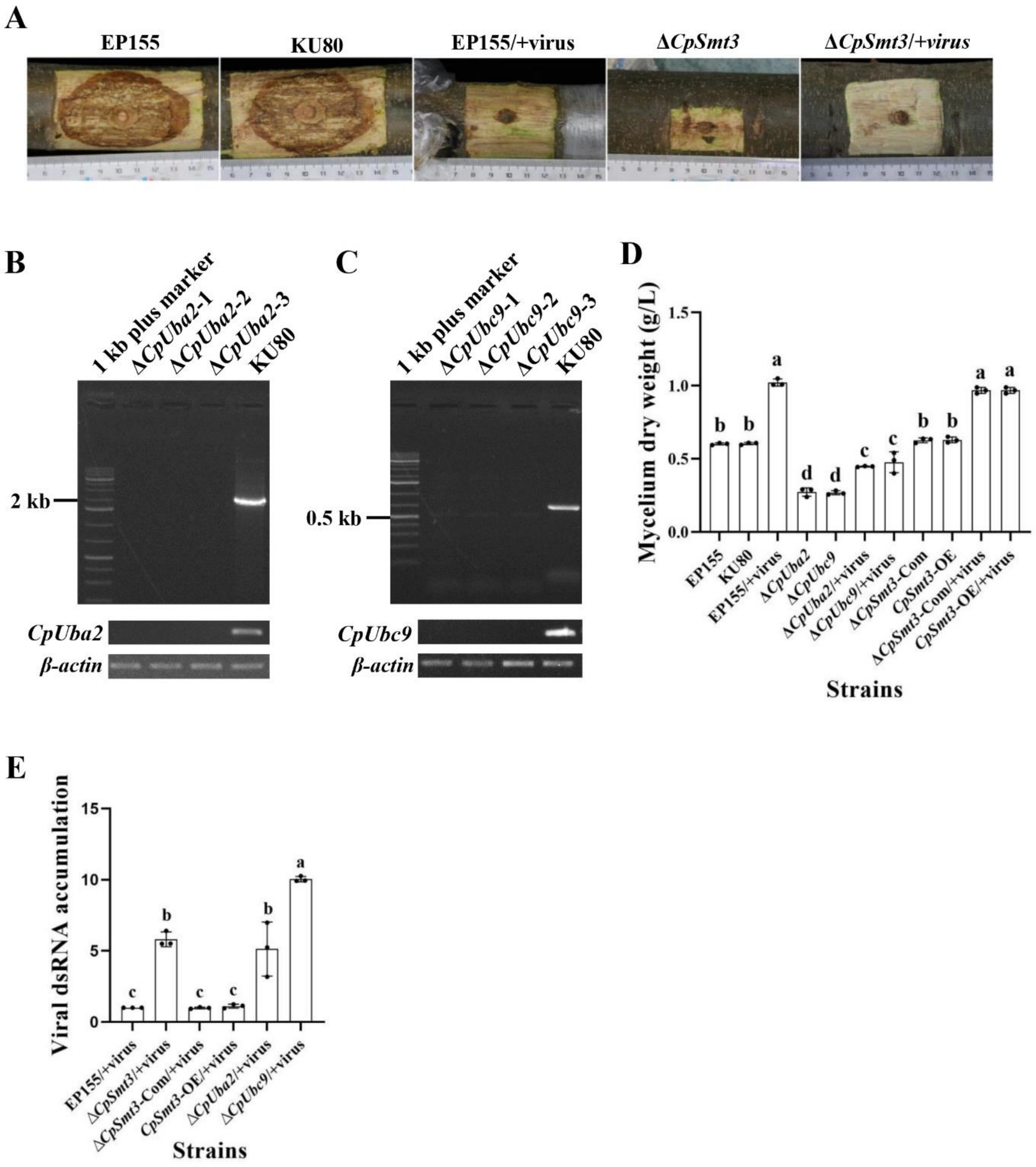

Figure S3. **(A)** Cankers induced by the tested strains on dormant stems of Chinese chestnut. **(B, C)** Identification of Δ*CpUba2* or Δ*CpUbc9* mutants at genomic and transcription level, respectively. The coding region of *CpUba2* is 2115 bp and the coding region of *CpUbc9* is 474 bp. The expected PCR and RT-PCR products of *CpUba2* are 2662 bp and 124 bp; the expected PCR and RT-PCR products of *Uba2* are 685 bp and 166 bp. **(D)** Mycelium dry weight statistics of the tested strains. **(E)** qRT-PCR quantification of viral dsRNA accumulation of the tested strains. The levels of dsRNA accumulation of indicated strains are represented as the fold change relative to that of the EP155/+virus. Error bars represent the standard deviation from three independent experiments. Different letters on the bars indicate significant differences ($p < 0.05$).

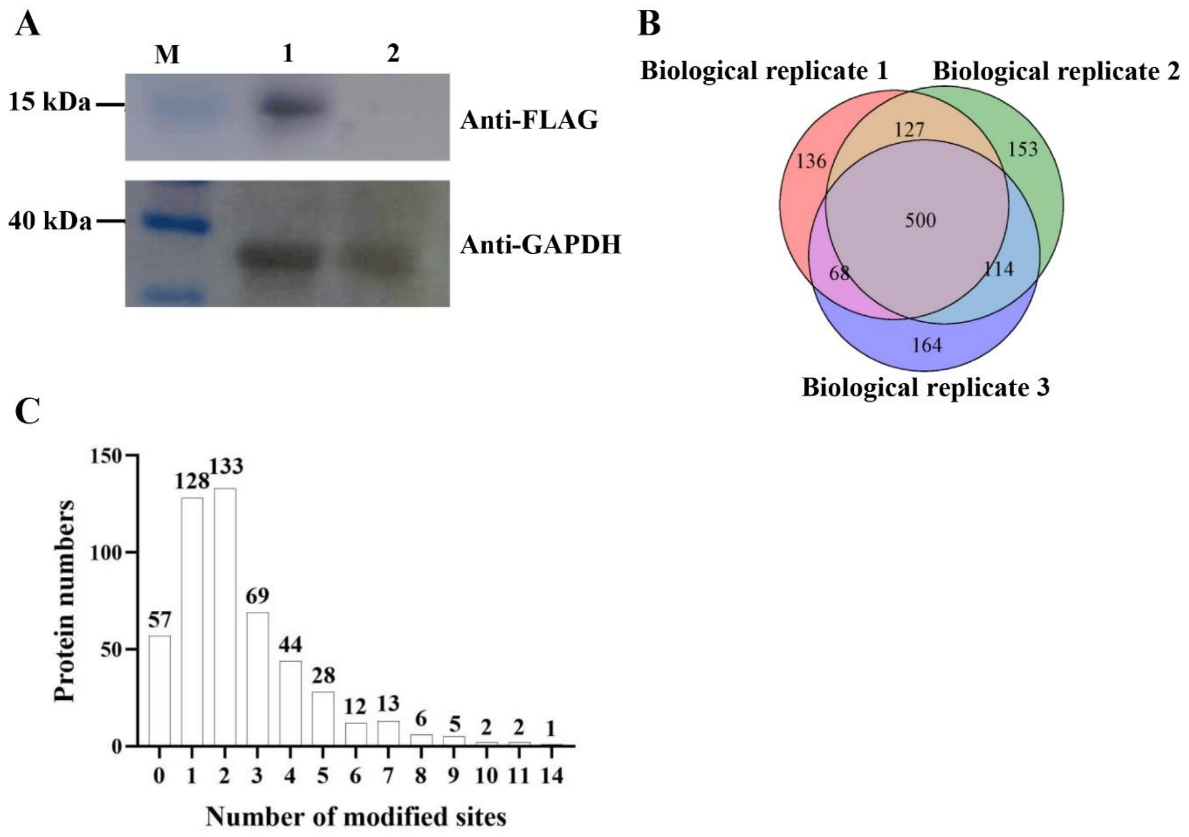

Figure S4. Identification of SUMO-modified proteins by affinity purification. **(A)** The expression of 3× FLAG-CpSmt3 in EP155 was verified by Western blot. Lane 1 was EP155/3× FLAG-CpSmt3 strain. Lane 2 was EP155. The total protein of EP155 was used as the control, and GAPDH was used as the internal reference. **(B)** Overlap of SUMO substrates from three biological replicates. **(C)** Distribution of SUMOylated proteins based on their number of modified sites.

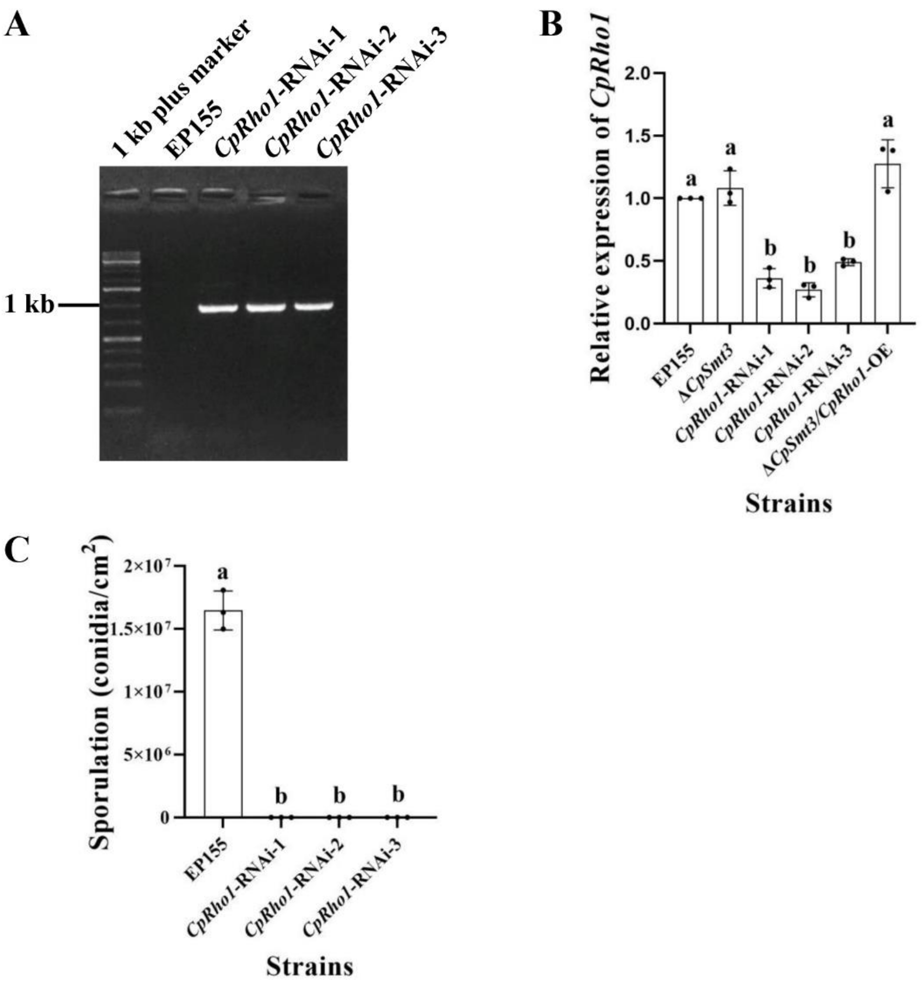

Figure S5. Construction of *CpRho1* RNA interference strain. **(A)** Identification of *CpRho1*-RNAi mutants by PCR. The strains that successfully introduce the RNA interfering vector can amplify 915 bp fragment. **(B)** The qRT-PCR analysis of the relative expression of *CpRho1* gene. The expression level of *CpRho1* in EP155 was set at 1.0. **(C)** Sporulation levels of the tested strains. Error bars represent the standard deviation from three independent experiments. Different letters on the bars indicate significant differences ($p < 0.05$).

Table S1 Primers used in this study.

Table S2 List of nonredundant proteins identified from proteomics of EP155 and Δ*CpSmt3*.

Table S3 List of SUMOylated proteins captured by affinity purification.

Table S4 GO enrichment analysis of down-regulated SUMOylated proteins in ΔCpSmt3

Table S5 KEGG enrichment analysis of down-regulated SUMOylated proteins in Δ*CpSmt3*.
